# Omental infarction and anterior wall adhesion presenting as surgical abdomen in a pediatric patient

**DOI:** 10.1002/ccr3.6604

**Published:** 2022-11-19

**Authors:** Kareem Omran, Wissam Jamal Al Tamr

**Affiliations:** ^1^ GKT School of Medical Education King's College London London UK; ^2^ NMC Royal (Al‐Zahra) Hospital Sharjah Sharjah United Arab Emirates

**Keywords:** acute abdomen, gastroenterology and hepatology, general surgery, pediatric surgery, pediatrics and adolescent medicine

## Abstract

Omental infarction is a rare cause of acute abdomen that can present in both the pediatric and adult populations causing adhesions or abscesses. Presentation may mimic appendicitis; however, ultrasonography may not be sufficient. We discuss the importance of CT imaging for the pre‐surgical diagnosis to avoid serious port‐site injuries.

## Graphical abstract

Omental infarction is a rare cause of acute abdomen that may mimic appendicitis. Complications such as adhesions may occur, and hence, CT imaging should be considered for the pre‐surgical diagnosis to avoid serious port‐site injuries.

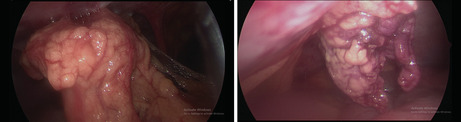

## 1 INTRODUCTION

Omental infarction is an uncommon cause of acute abdomen often mimicking the presentation of acute appendicitis in the pediatric population.^1^ It occurs in 0.1%–0.5% of children undergoing surgery for suspected appendicitis.^2^ Omental infarction is caused by two main pathological mechanisms: either secondary to vascular pedicle torsion on its own axis, or due to hypercoagulable states. One third of torsion cases can be idiopathic, with two thirds being due to the presence of intra‐abdominal pathology causing distal anchorage of the omentum.^3^ It has been shown that obesity is a risk factor for this disease.^4^

This article describes the management of a case of omental infarction in a 6‐year‐old patient presenting to the hospital with an acute surgical abdomen and discusses the condition's relevance in pediatric surgery. The need for awareness about the condition and preoperative imaging is emphasized.

## 2 CASE HISTORY/EXAMINATION

A 6‐year‐old patient presented to the hospital with acute abdominal pain and constipation, having passed dry, hard motions every 3 days. He was given analgesia and Daflon (450 mg diosmin, 50 mg hesperidin) in hopes of relieving the symptoms and was discharged. Having severely deteriorated, he then re‐presented to the hospital a week later with a surgical abdomen (rigid and tender with guarding). The patient had a BMI of 22.22, putting him in the 99th centile for age, and classed as “very overweight.” The patient has no surgical history. Differentials that were considered included a small bowel obstruction; however, the patient had no emesis. Further, the absence of fever and a loss of appetite also discouraged a diagnosis of appendicitis.

## 3 INVESTIGATIONS

Routine bloods were acquired prior to surgery showing an elevated white blood cell count (WBC) of 17.8 × 10^9^/L and neutrophilia with 85.7% neutrophils. CRP was also elevated at 35.

**FIGURE 1:**
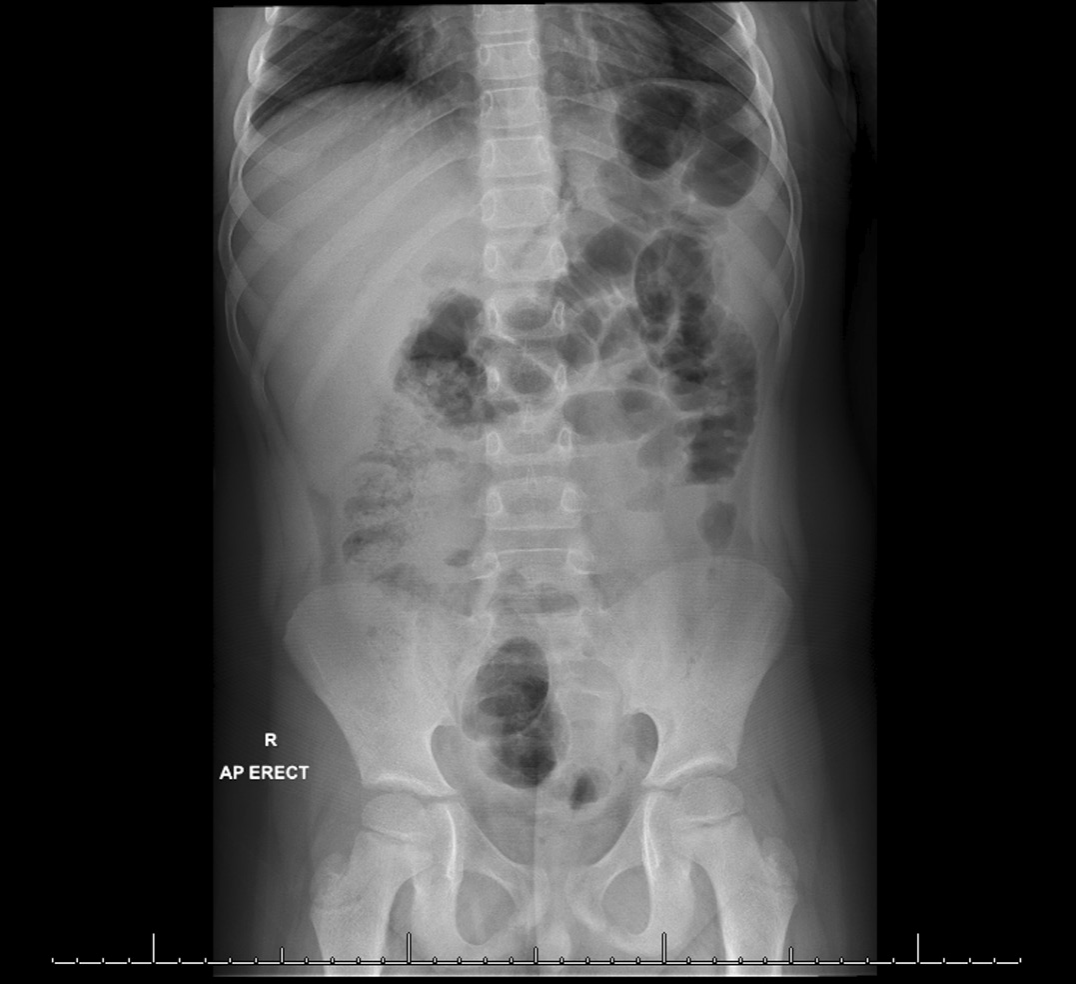
Supine abdominal X‐ray which was incapable of picking up the omental mass that was infarcted at the umbilical level.

**FIGURE 2:**
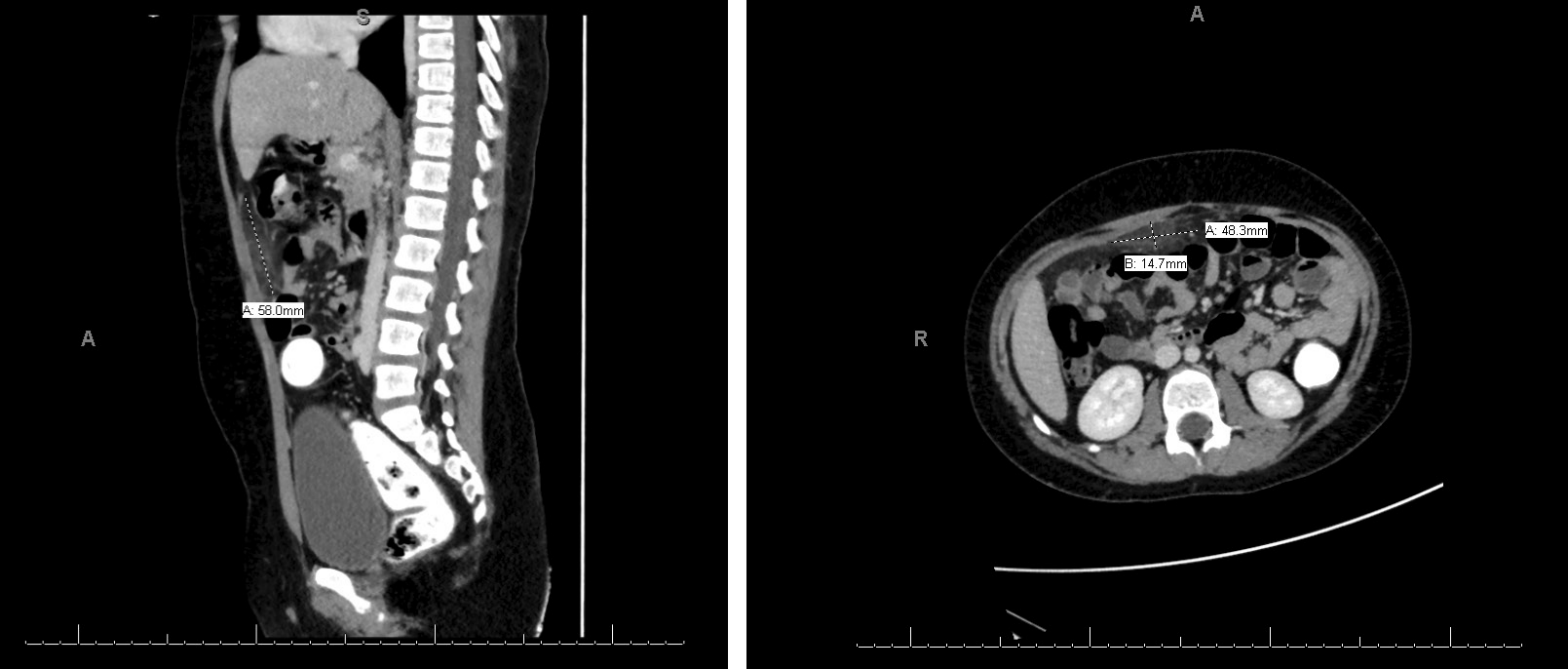
Sagittal and transverse CT scans showing an adherent omental mass on the anterior abdominal wall measuring approximately 5.5 × 1.7 cm.

An ultrasound was requested on suspect of appendicitis; however, it was deemed inconclusive. Further, an abdominal X‐ray was requested which showed fecal and gaseous distension of the large bowel with no evidence of free air under the diaphragms. There was no radio‐opaque calculus or abnormal calcification, which did not lead to any conclusive pathology (Figure 1). Prior to undergoing explorative laparoscopic laparotomy, computed tomography (CT) imaging was utilized in hopes of discovering the pathology. Here, the CT showed an ill‐defined region of ground glass haziness involving the omentum in the anterior supraumbilical region measuring 5.5 × 1.7 cm (Figure 2). Moreover, the presence of multiple enlarged and sub‐centimeter sized mesenteric lymph nodes pointed toward a diagnosis of omental necrosis.

## 4 TREATMENT

The patient was then taken for laparoscopic surgery where port sites were carefully chosen to avoid an umbilical insertion as to not pierce the adherent omentum. Open technique was used to enter the peritoneal cavity. The laparoscope was then inserted into the abdomen under direct vision, and a 10 mm port was inserted. Subsequently, the following ports were inserted under direct visualization along with local anesthetic in the typical fashion: a left lower quadrant 5 mm port and a 5 mm right lower quadrant port.

**FIGURE 3:**
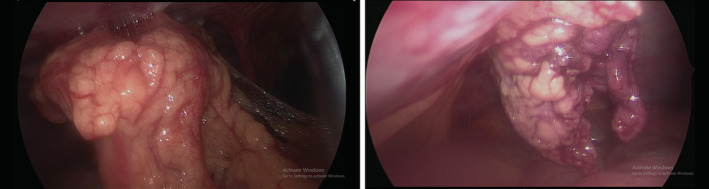
Image from the laparoscope showing the adherent omental mass on the anterior abdominal wall, at the umbilical level.

**FIGURE 4:**
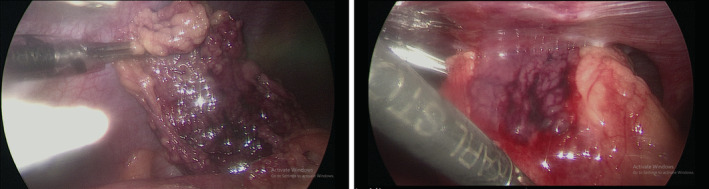
Image from the laparoscope showing the necrosed omental mass with severe congestion.

After a general inspection of the organs and the abdomen, the omental adhesion was carefully released from the umbilicus (Figure 3). It was noted that there was severe congestion of the large omental mass. It was also necrosed and twisted once round, suggesting primary idiopathic torsion of the omentum as the etiology (Figure 4). The omental mass was then resected and sent to histopathology, which showed mature adult‐type adipose tissue with acute and chronic inflammation, granulation tissue, dilated vessels, and fibrosis. No significant increase in pleomorphism or mitosis was seen. This confirmed the diagnosis of omental necrosis secondary to infarction. Postoperatively, the patient assumed spontaneous recovery. The WBC returned to normal levels within 2 days, and the patient was discharged.

## 5 DISCUSSION

Omental infarction is a serious condition that can be easily misdiagnosed, as the clinical presentation is similar to other causes of acute abdomen such as appendicitis. Although it is a rare condition, the importance lies in the need of adequate imaging and diagnosis prior to surgery, as we have shown how omental infarction may lead to, or be concomitant with anterior abdominal wall adhesions.^5^ Irreflective laparoscopic laparotomy in said cases may cause penetration of the omental adhesion, resulting in heavy bleeding in the patient.^6^

Though it is believed that conservative management may be sufficient in some cases, our case showed that it resulted in deterioration of the patient's state, and hence, surgical intervention was necessary. Moreover, it has been shown that both a younger age and an elevated white blood count ≥12 × 10^9^/L were predictive of conservative treatment failure—which our case has reaffirmed.^3^ Surgical intervention has also been shown to reduce length of hospital stay, with patients staying for 2.5 days as opposed to 5.1.^3^ The feared complication in the conservative management of omental infarction is the development of an omental abscess, which can lead to severe deterioration of the patient and peritonitis.^7^ As such we recommend that in the pediatric population, the need for surgical intervention should not be neglected.

Some literature suggests ultrasound as the modality of choice in the diagnosis and management of omental infarction^8^; however, another study has shown it to have a sensitivity of 64%.^9^ Moreover, the operator dependent nature of ultrasonography and lack of awareness of the condition limit its success.^10^ In our case, the ultrasonographer may not have had omental infarction with adhesions to the abdominal wall as a differential, and hence may not have investigated the appropriate area. Findings which can be present on ultrasonography can include a complex mass, a mixture of solid material, and hypoechoic zones^11^—however, this was not found on our report. As such, while ultrasonography should be used as initial imaging to exclude obvious causes of acute abdomen such as appendicitis, if inconclusive, CT should be followed. CT imaging has a much greater sensitivity of around 90%, and its use in cases of acute abdomen has resulted in the ability to perform a perioperative diagnosis much more often.^9^, ^12^

## 6 CONCLUSION

In conclusion, while there have been previous case reports on omental infarction mispresenting as appendicitis, our case indicates the importance of the consideration of surgical intervention in the pediatric population, as well as the necessity for preoperative diagnosis prior to laparoscopic laparotomy to ensure port‐site injury and heavy omental bleeding is avoided.

## AUTHOR CONTRIBUTIONS

Kareem Omran (First author): Write up of the manuscript as well as literature review. Dr. Wissam Jamal Al Tamr (Senior author) served as a senior physician who operated the case and supervised the write up.

## FUNDING INFORMATION

No sources of support or external funding.

## CONFLICT OF INTEREST

We disclose no conflict of interest.

## CONSENT

Written informed consent was obtained from the patient to publish this report in accordance with the journal's patient consent policy.

## Data Availability

Data sharing not applicable to this article as no datasets were generated or analysed during the current study.
